# Supplementary material for: Systematic Review and Modelling of Age-Dependent Prevalence of Toxoplasma gondii in Livestock, Wildlife and Felids in Europe
Source: Pathogens. 2023 Jan 6;12(1):97. doi: 10.3390/pathogens12010097 (PMC9865579; doi:10.3390/pathogens12010097)
Supplement: Supplementary file 1 [file pathogens-12-00097-s001.zip › Suplementary figures.pdf]

Supplementary figures to the article: "Systematic review and modelling of age-dependent prevalence of *Toxoplasma gondii* in livestock, wildlife and felids in Europe"

Content of the file: Figure S1: Search string; Figure S2: PRISMA flow diagram; Figure S3: Trace plots; Figure S4: Hyperparameters; Figure S5: Modelled seroprevalence – chicken, hen, turkey; Figure S6: Modelled seroprevalence – duck, goose; Figure S7: Modelled seroprevalence – felids; Figure S8: Modelled seroprevalence – goat; Figure S9: Modelled seroprevalence – lagomorphs; Figure S10: Modelled seroprevalence – pig; Figure S11: Modelled seroprevalence – sheep; Figure S12: Modelled seroprevalence – wild birds; Figure S13: Modelled seroprevalence – wild boar; Figure S14: Modelled seroprevalence – wild ruminants; Figure S15: Age shift.

#48	#20 OR #47	1,984
#47	#46 AND ('article'/it OR 'article in press'/it OR 'review'/it)	1,380
#46	#44 OR #45	1,537
#45	#43 NOT #16	1,440
#44	#8 AND #43	521
#43	#5 AND #42	2,499
#42	#21 OR #22 OR #23 OR #24 OR #25 OR #26 OR #27 OR #28 OR #29 OR #30 OR #31 OR #32 OR #33 OR #34 OR #35 OR #36 OR #37 OR #38 OR #39 OR #40 OR #41	1,690,912
#41	'meat'/exp OR beef:ti,ab OR mutton:ti,ab OR pork:ti,ab OR veal:ti,ab OR 'red meat':ti,ab	76,206
#40	'anas platyrhynchos'/exp OR 'mallard*':ti,ab	2,074
#39	'grouse'/exp OR 'grouse*':ti,ab OR 'ptarmigan*':ti,ab OR 'lagopus*':ti,ab	1,098
#38	'deer'/exp OR deer:ti,ab OR 'reindeer*':ti,ab OR moose:ti,ab	17,299
#37	'leporidae'/exp OR 'rabbit*':ti,ab OR hare:ti,ab	395,732
#36	'quail'/exp OR 'quail*':ti,ab	10,889
#35	'guineafowl'/exp OR 'guineafowl*':ti,ab	157
#34	'perdicinae'/exp OR 'partridge*':ti,ab OR 'pheasant*':ti,ab	4,488
#33	'columbidae'/exp OR 'pigeon*':ti,ab	17,387
#32	'phasianidae'/exp/mj	37,945
#31	'phasianus colchicus'/exp OR 'phasianus colchicus':ti,ab	339
#30	'duck'/exp OR duck:ti,ab OR ducks:ti,ab	15,297

#29	'goose'/exp OR goose:ti,ab OR geese:ti,ab	5,001
#28	'chicken'/exp OR 'chicken*':ti,ab	172,135
#27	'turkey bird'/exp	10,757
#26	'equus'/exp OR equus:ti,ab OR 'horse*':ti,ab OR mule:ti,ab OR 'donkey*':ti,ab	125,086
#25	'goat'/exp OR 'goat*':ti,ab	45,663
#24	'sheep'/exp OR sheep:ti,ab OR 'lamb*':ti,ab OR 'mouflon*':ti,ab	186,222
#23	'poultry'/exp OR 'poultry*':ti,ab	43,252
#22	'pig'/exp OR pig:ti,ab OR pigs:ti,ab OR 'wild boar*':ti,ab	354,175
#21	'bovine'/exp OR bovine:ti,ab OR cattle:ti,ab OR buffalo:ti,ab OR cow:ti,ab OR cows:ti,ab OR calf:ti,ab OR calves:ti,ab	470,320
#20	#19 AND ('article'/it OR 'article in press'/it OR 'review'/it)	816
#19	#17 OR #18	973
#18	#7 NOT #16	899
#17	#7 AND #8	298
#16	#9 OR #10 OR #11 OR #12 OR #13 OR #14 OR #15	3,674,850
#15	'brazil*':ti OR 'russia*':ti OR 'australia*':ti OR 'japan*':ti OR china:ti OR chinese:ti OR 'colombi*':ti OR 'iran*':ti OR galapagos:ti OR 'korea*':ti OR 'saudi arabia*':ti OR 'mexic*':ti OR 'india*':ti OR 'indonesia*':ti OR peru:ti OR iraq:ti OR mongolia:ti OR thailand:ti OR 'pakistan*':ti OR 'angol*':ti OR 'south africa*':ti OR usa:ti OR 'united states':ti OR 'algeria*':ti OR qatar:ti OR kenya:ti OR 'ethiopia*':ti	985,492
#14	'arctic and antarctic'/exp OR arctic:ti OR antarctic:ti	17,602
#13	'oceanic regions'/exp	113,080
#12	'western hemisphere'/exp	1,733,501
#11	'australia and new zealand'/exp OR 'australia*':ti OR 'new zealand':ti	248,231
#10	'asia'/exp OR asia:ti	1,061,865

#9	'africa'/exp OR 'africa*':ti	384,849
#8	'europe'/exp OR 'europ*':ti,ab OR france:ti,ab OR french:ti,ab OR 'german*':ti,ab OR netherlands:ti,ab OR dutch:ti,ab OR 'belgi*':ti,ab OR spain:ti,ab OR spanish:ti,ab OR italy:ti,ab OR italian:ti,ab OR switzerland:ti,ab OR swiss:ti,ab OR 'austria*':ti,ab OR 'bulgaria*':ti,ab OR 'hungar*':ti,ab OR ireland:ti OR irish:ti,ab OR 'united kingdom':ti,ab OR 'croatia*':ti,ab OR poland:ti,ab OR polish:ti,ab OR 'czech*':ti,ab OR portugal:ti,ab OR 'portugues*':ti,ab OR liechtenstein:ti,ab OR luxembourg:ti,ab OR denmark:ti,ab OR danish:ti,ab OR finland:ti,ab OR finnish:ti,ab OR iceland:ti,ab OR norway:ti,ab OR sweden:ti,ab OR swedish:ti,ab OR estonia:ti,ab OR 'latvia*':ti,ab OR 'lithuania*':ti,ab OR 'moldova*':ti,ab OR 'romania*':ti,ab OR 'slovakia*':ti,ab OR ukraine:ti,ab OR belarus:ti,ab OR andorra:ti,ab OR malta:ti,ab OR monaco:ti,ab OR 'san marino':ti,ab OR 'albania*':ti,ab OR 'bosnia and herzegovina':ti,ab OR bosnian:ti,ab OR cyprus:ti,ab OR greece:ti,ab OR 'greek*':ti,ab OR 'kosovo*':ti,ab OR 'republic of macedonia':ti,ab OR montenegro:ti,ab OR 'serbia*':ti,ab OR 'slovenia*':ti,ab OR corsica:ti,ab OR madeira:ti,ab OR azores:ti,ab OR gibraltar:ti,ab OR sardinia:ti,ab OR sicily:ti,ab OR 'canary island*':ti,ab OR 'channel island*':ti,ab OR guernsey:ti,ab OR jersey:ti,ab OR 'isle of	2,611,070

	man':ti,ab OR 'scandinavia*':ti,ab OR 'faroe islands':ti,ab OR england:ti,ab OR scotland:ti,ab OR wales:ti,ab OR 'great britain':ti,ab OR mallorca:ti,ab OR menorca:ti,ab OR ibiza:ti,ab OR formentera:ti,ab OR tenerife:ti,ab OR fuerteventura:ti,ab OR 'gran canaria':ti,ab OR lanzarote:ti,ab OR 'la palma':ti,ab OR 'la gomera':ti,ab OR 'el hierro':ti,ab OR 'la graciosa':ti,ab OR aland:ti,ab	
#7	#5 AND #6	1,654
#6	'felidae'/exp OR cat:ti,ab OR cats:ti,ab OR 'lynx*':ti,ab OR 'felis silvestris'/exp OR 'felis silvest*':ti,ab OR 'wildcat*':ti,ab OR 'wild-cat*':ti,ab	225,235
#5	(#1 OR #2 OR #3) AND [2000-2020]/py	21,086
#4	#1 OR #2 OR #3	37,210
#3	'toxoplasma'/exp AND 'gondii*':ti,ab	13,792
#2	'toxoplasma gondii'/exp OR (('toxoplasma*' NEAR/2 'gondii*'):ti,ab)	20,176
#1	'toxoplasmosis'/exp OR 'toxoplasmos*':ti,ab	28,413

Figure S1: Search string. Search terms, with the use of Emtree terms, applied in Embase database in the search of articles for the systematic literature review. Search groups in the search are numbered and provided is a number of corresponding results found for each search group (column on the right).

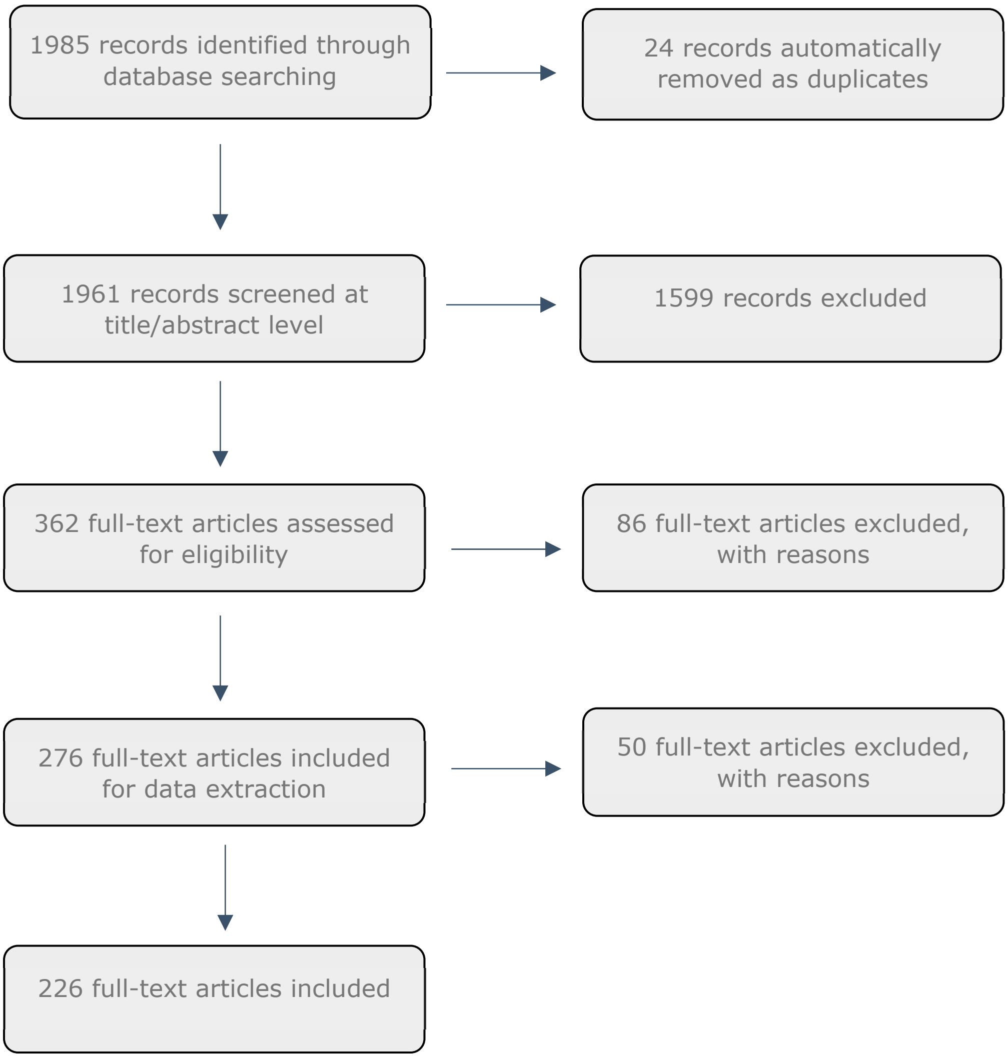

Figure S2: PRISMA flow diagram. The flow diagram represents graphically the screening steps and data extraction step with numbers of publications excluded and number of publications proceeding to the following step, leading to the final number of included articles.

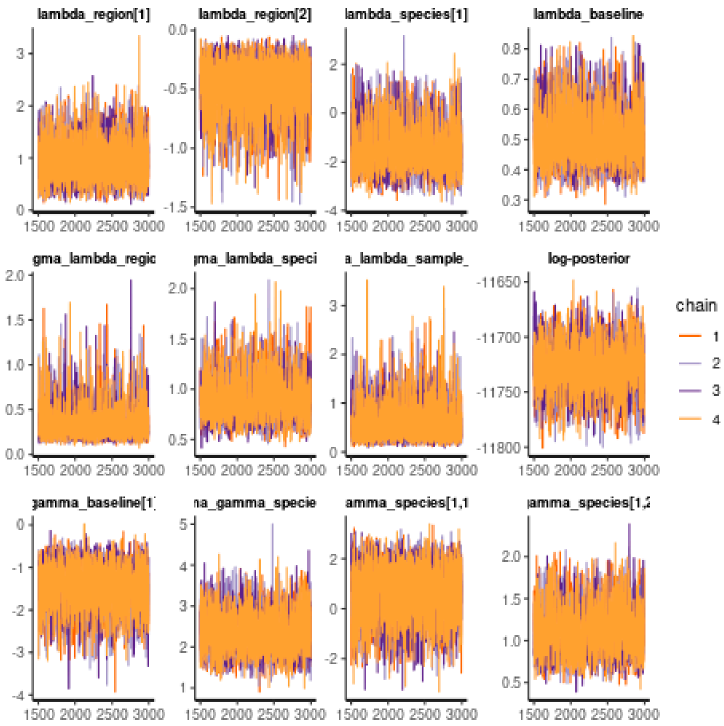

Figure S3: Trace plots. Posterior densities of individual parameters of prior distributions (hyperparameters) for the forces of infection and reversion rates were visualised in panels (for hyperparameters see Supplementary files - Figure S4), showing the clustering of data.

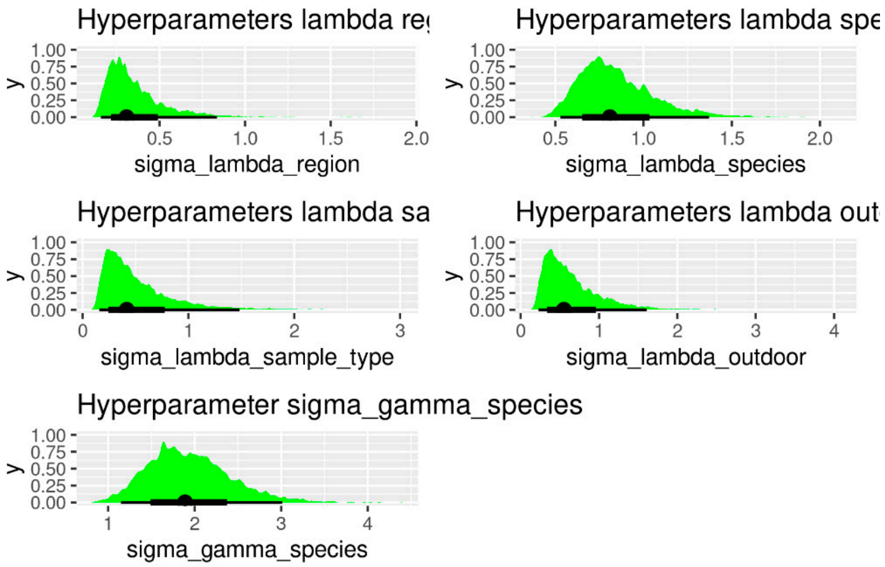

Figure S4: Hyperparameters. Posterior densities of the hyperparameters in the model. The uncertainty interval is shown as the grey area in the figures and the thin and thick black lines on the x axis indicate 95% and 50% Bayesian credible intervals, respectively, with a dot indicating the mean. The information about the confidence intervals is also supplied numerically in the boxes. Higher values indicate higher heterogeneity.

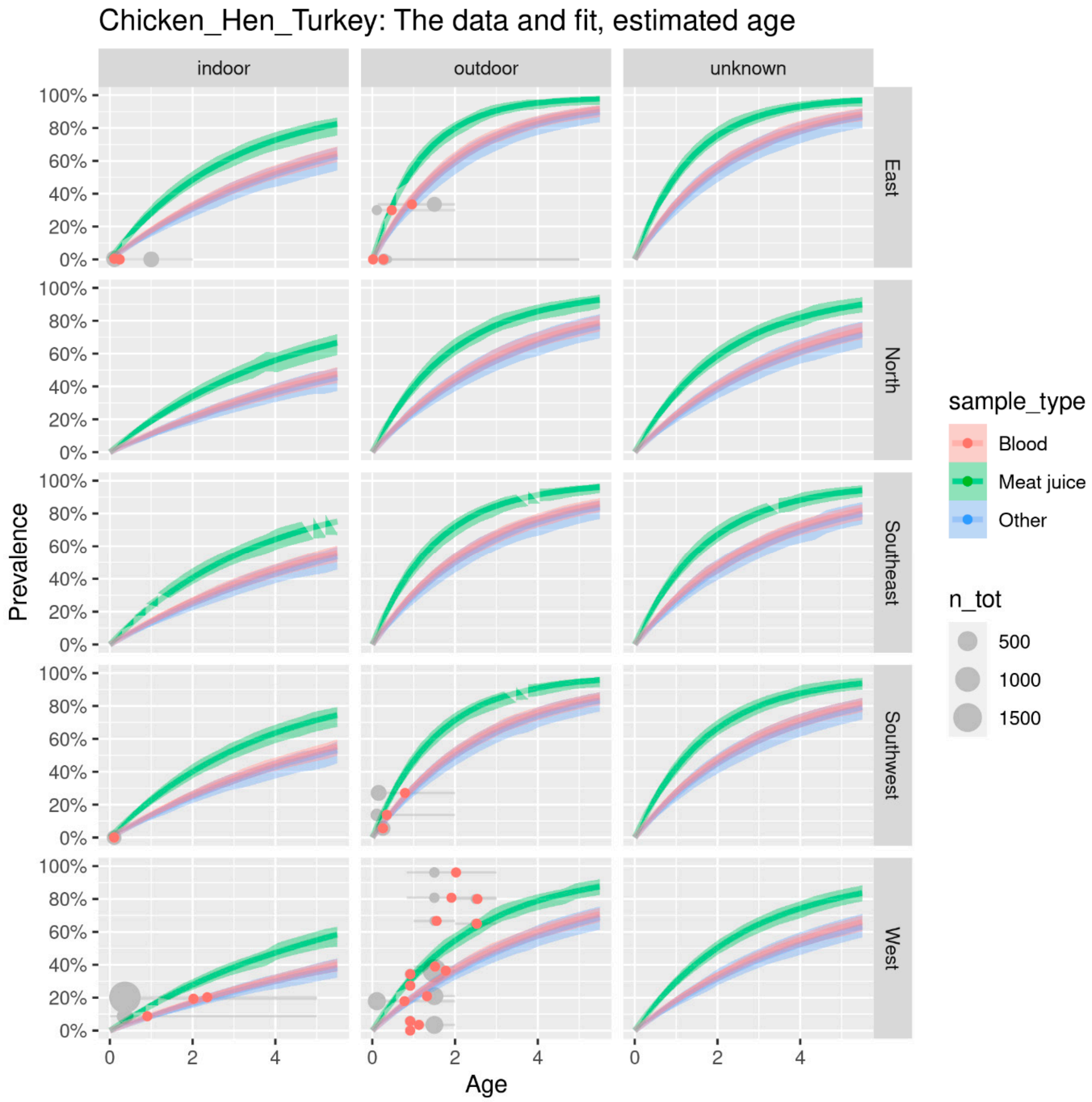

Figure S5: Modelled seroprevalence – chicken, hen, turkey. SIS model fit for age-dependent seroprevalence of *T. gondii* in poultry (chicken, hen, turkey) by outdoor access in five regions of Europe. The lines indicate the fitted seroprevalence by age, based on detection in blood (red), muscle fluid (green) or other matrices (blue). The grey dots represent seroprevalence data points at the best estimate of age in the data for the studied populations. The dots are shifted horizontally along the grey line extending from the minimum to maximum possible age, to the best fitting age (red dots) (see Supplementary files – Fig. S15).

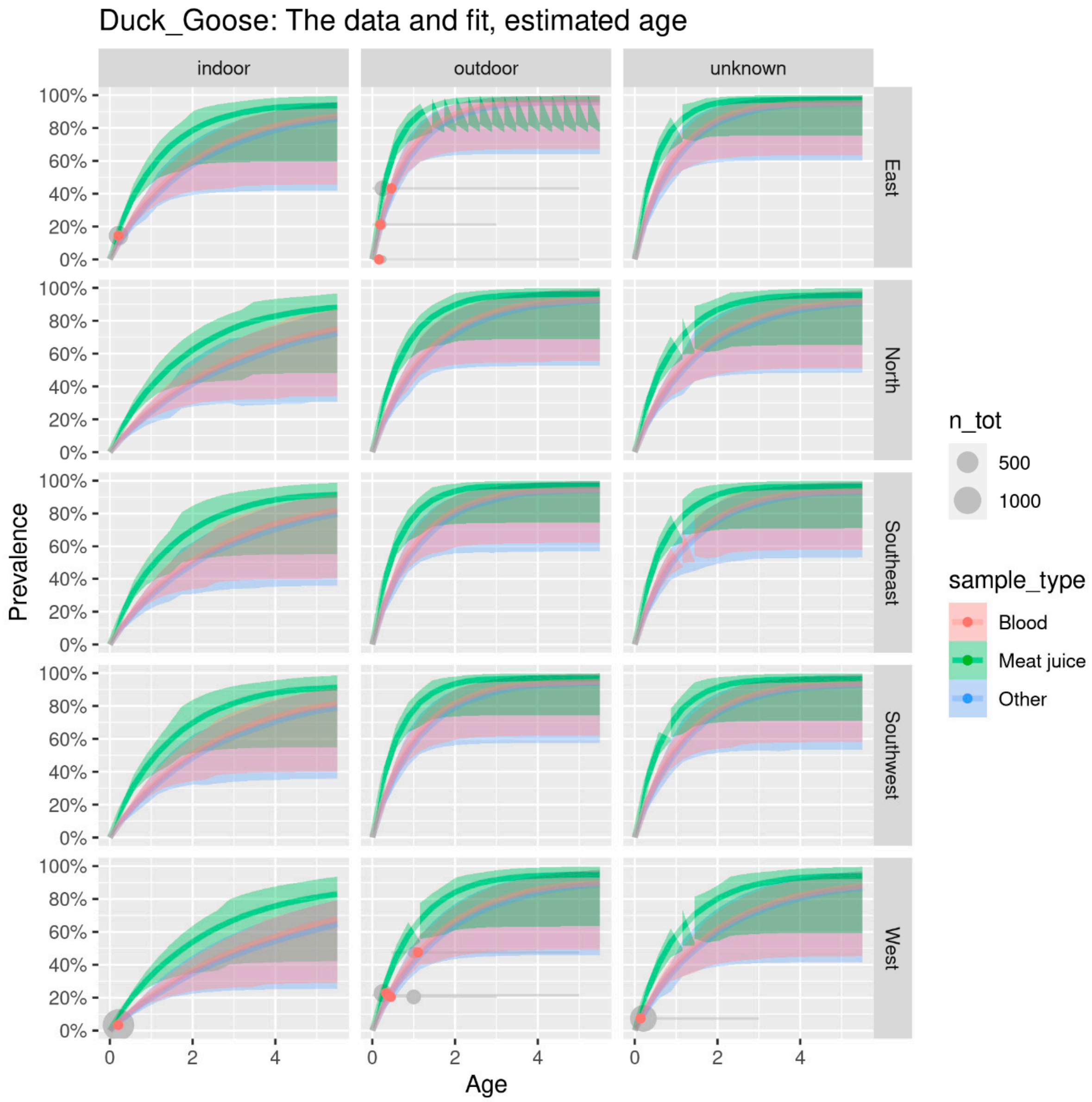

Figure S6: Modelled seroprevalence – duck, goose. SIS model fit for age-dependent seroprevalence of *T. gondii* in ducks and geese by outdoor access in five regions of Europe. The lines indicate the fitted seroprevalence by age, based on detection in blood (red), muscle fluid (green) or other matrices (blue). The grey dots represent seroprevalence data points at the best estimate of age in the data for the studied populations. The dots are shifted horizontally along the grey line extending from the minimum to maximum possible age, to the best fitting age (red dots) (see Supplementary files – Fig. S15).

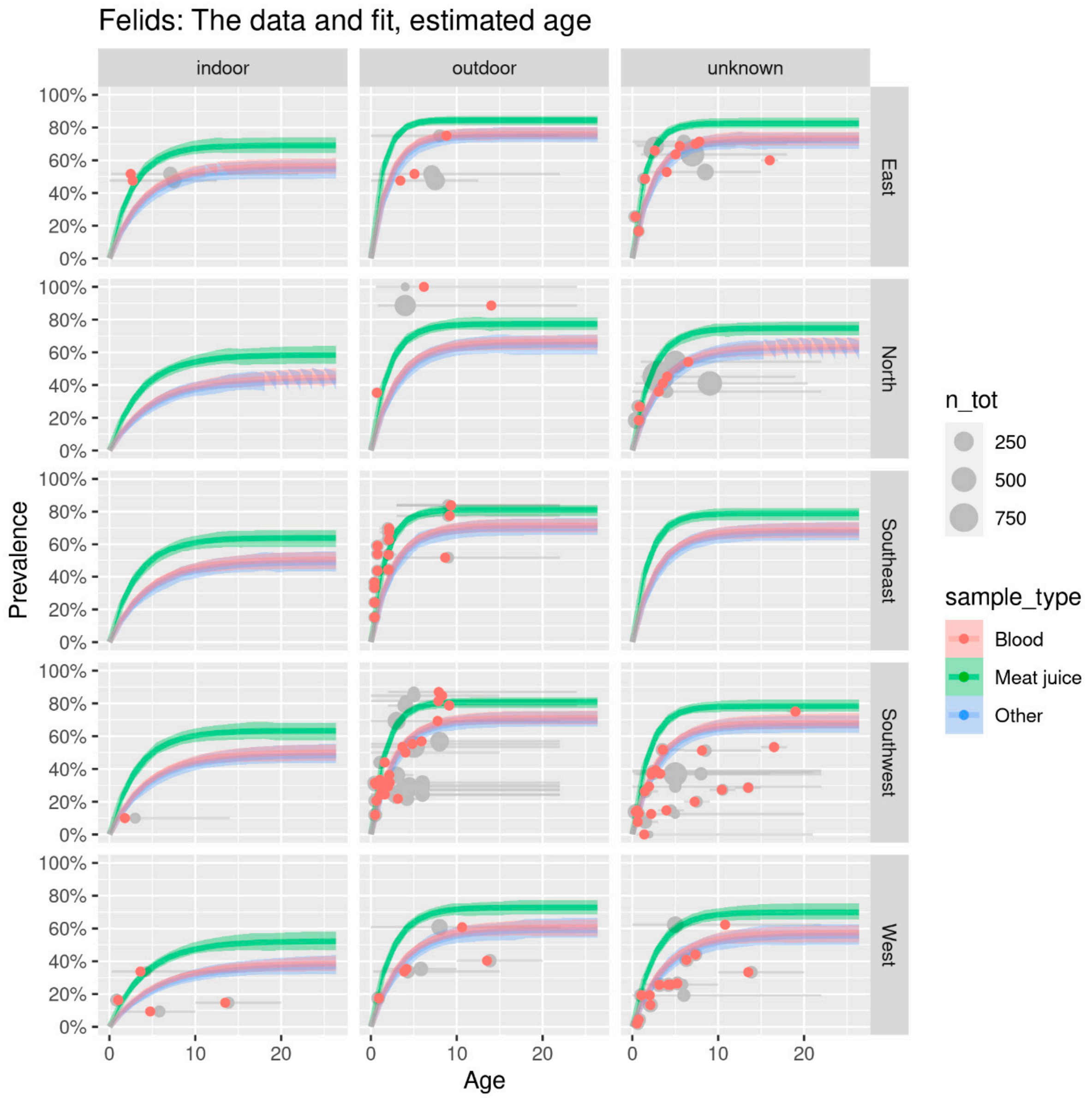

Figure S7: Modelled seroprevalence – felids. SIS model fit for age-dependent seroprevalence of *T. gondii* in felids by outdoor access in five regions of Europe. The lines indicate the fitted seroprevalence by age, based on detection in blood (red), muscle fluid (green) or other matrices (blue). The grey dots represent seroprevalence data points at the best estimate of age in the data for the studied populations. The dots are shifted horizontally along the grey line extending from the minimum to maximum possible age, to the best fitting age (red dots) (see Supplementary files – Fig. S15).

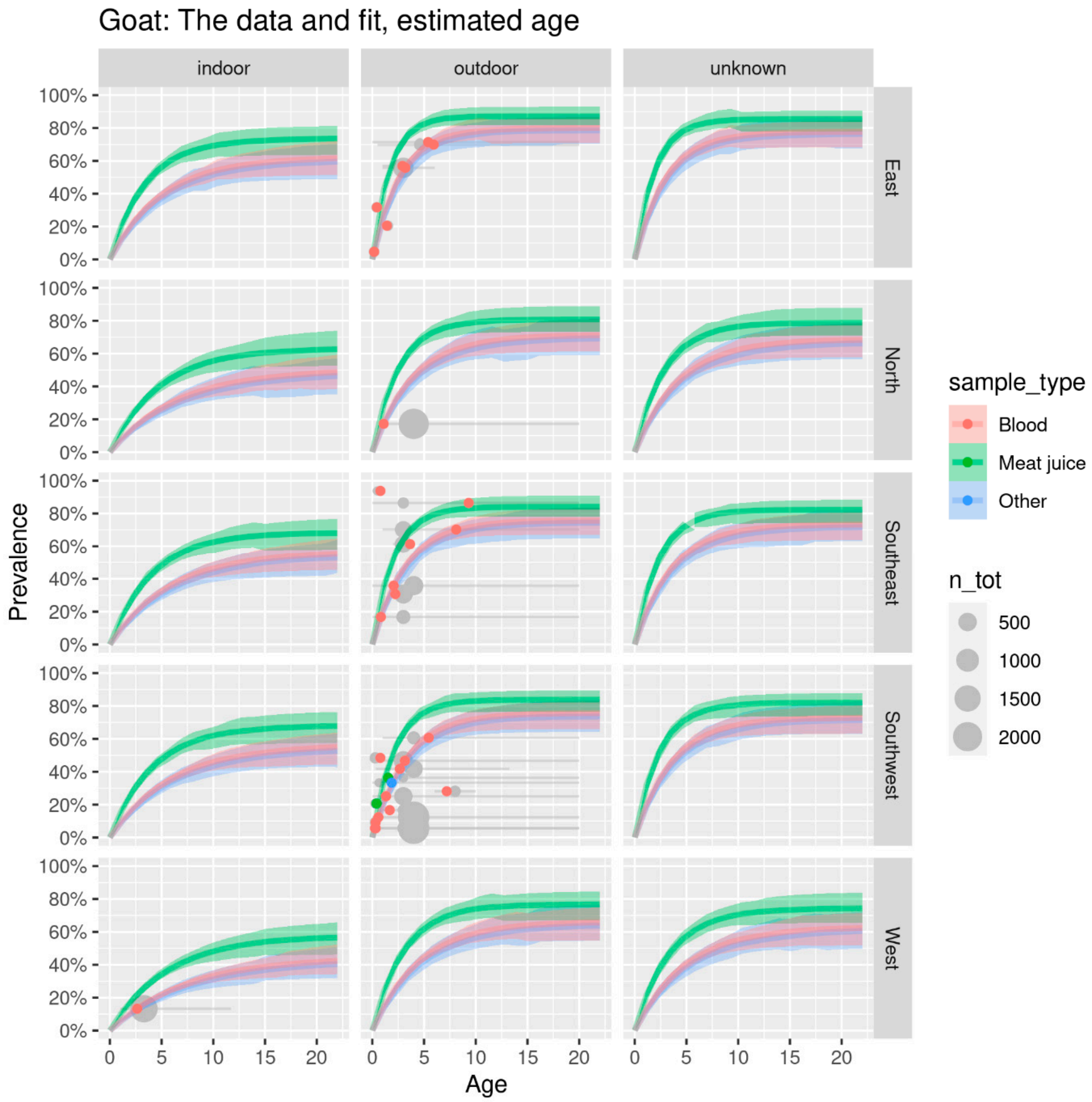

Figure S8: Modelled seroprevalence – goat. SIS model fit for age-dependent seroprevalence of *T. gondii* in goats by outdoor access in five regions of Europe. The lines indicate the fitted seroprevalence by age, based on detection in blood (red), muscle fluid (green) or other matrices (blue). The grey dots represent seroprevalence data points at the best estimate of age in the data for the studied populations. The dots are shifted horizontally along the grey line extending from the minimum to maximum possible age, to the best fitting age (red dots) (see Supplementary files – Fig. S15).

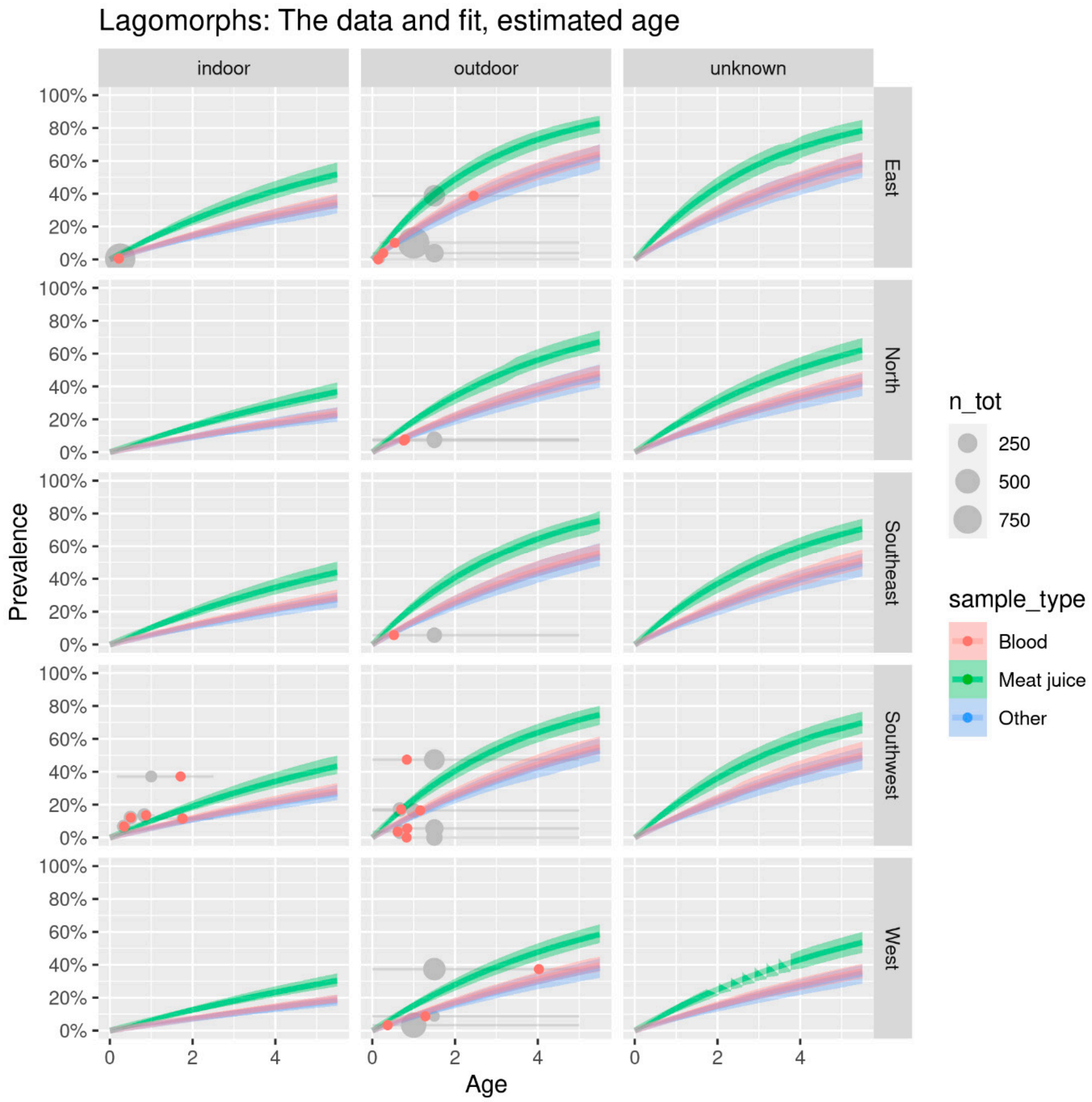

Figure S9: Modelled seroprevalence – lagomorphs. SIS model fit for age-dependent seroprevalence of *T. gondii* in lagomorphs by outdoor access in five regions of Europe. The lines indicate the fitted seroprevalence by age, based on detection in blood (red), muscle fluid (green) or other matrices (blue). The grey dots represent seroprevalence data points at the best estimate of age in the data for the studied populations. The dots are shifted horizontally along the grey line extending from the minimum to maximum possible age, to the best fitting age (red dots) (see Supplementary files – Fig. S15).

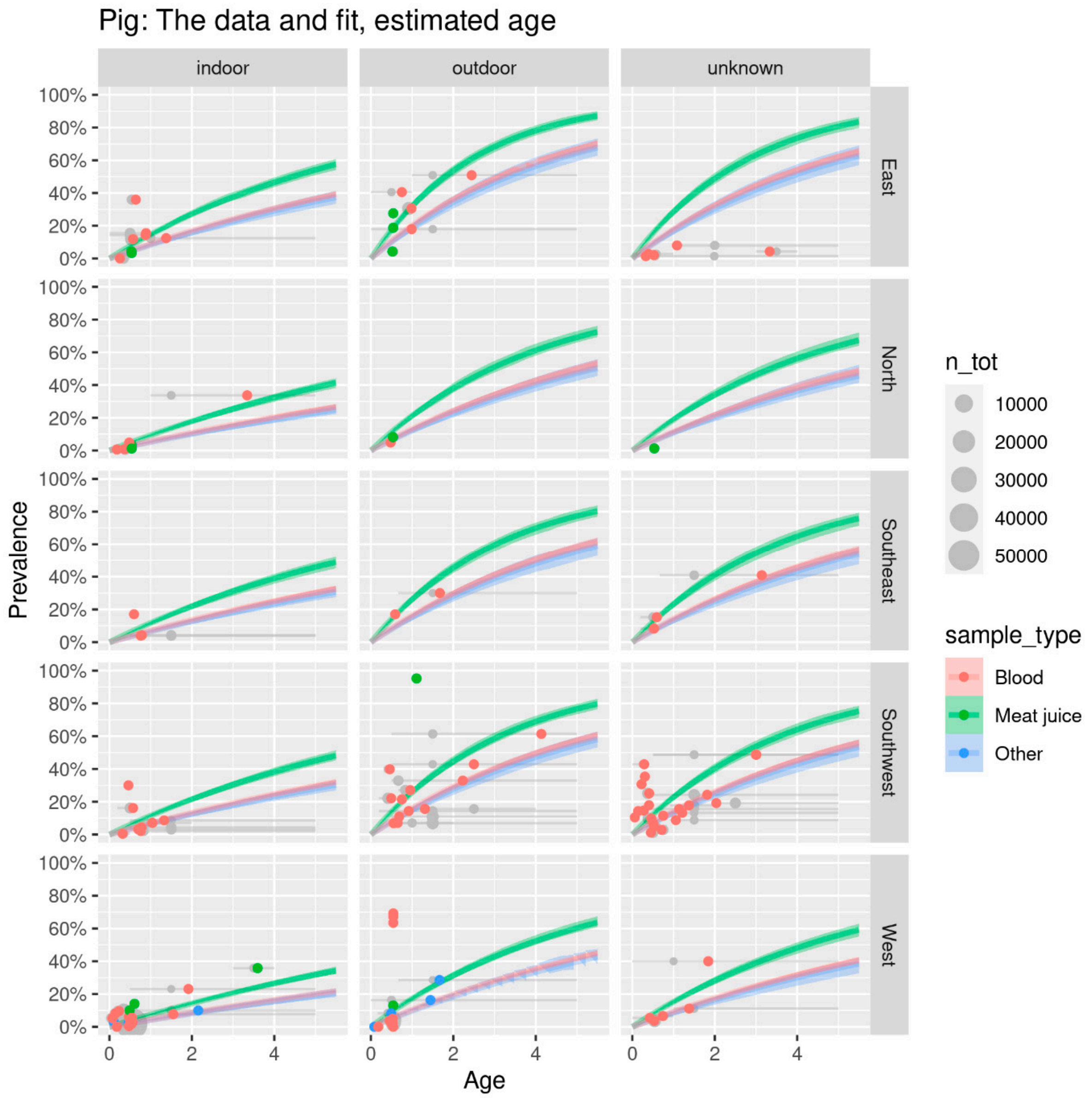

Figure S10: Modelled seroprevalence – pig. SIS model fit for age-dependent seroprevalence of *T. gondii* in pigs by outdoor access in five regions of Europe. The lines indicate the fitted seroprevalence by age, based on detection in blood (red), muscle fluid (green) or other matrices (blue). The grey dots represent seroprevalence data points at the best estimate of age in the data for the studied populations. The dots are shifted horizontally along the grey line extending from the minimum to maximum possible age, to the best fitting age (red dots) (see Supplementary files – Fig. S15).

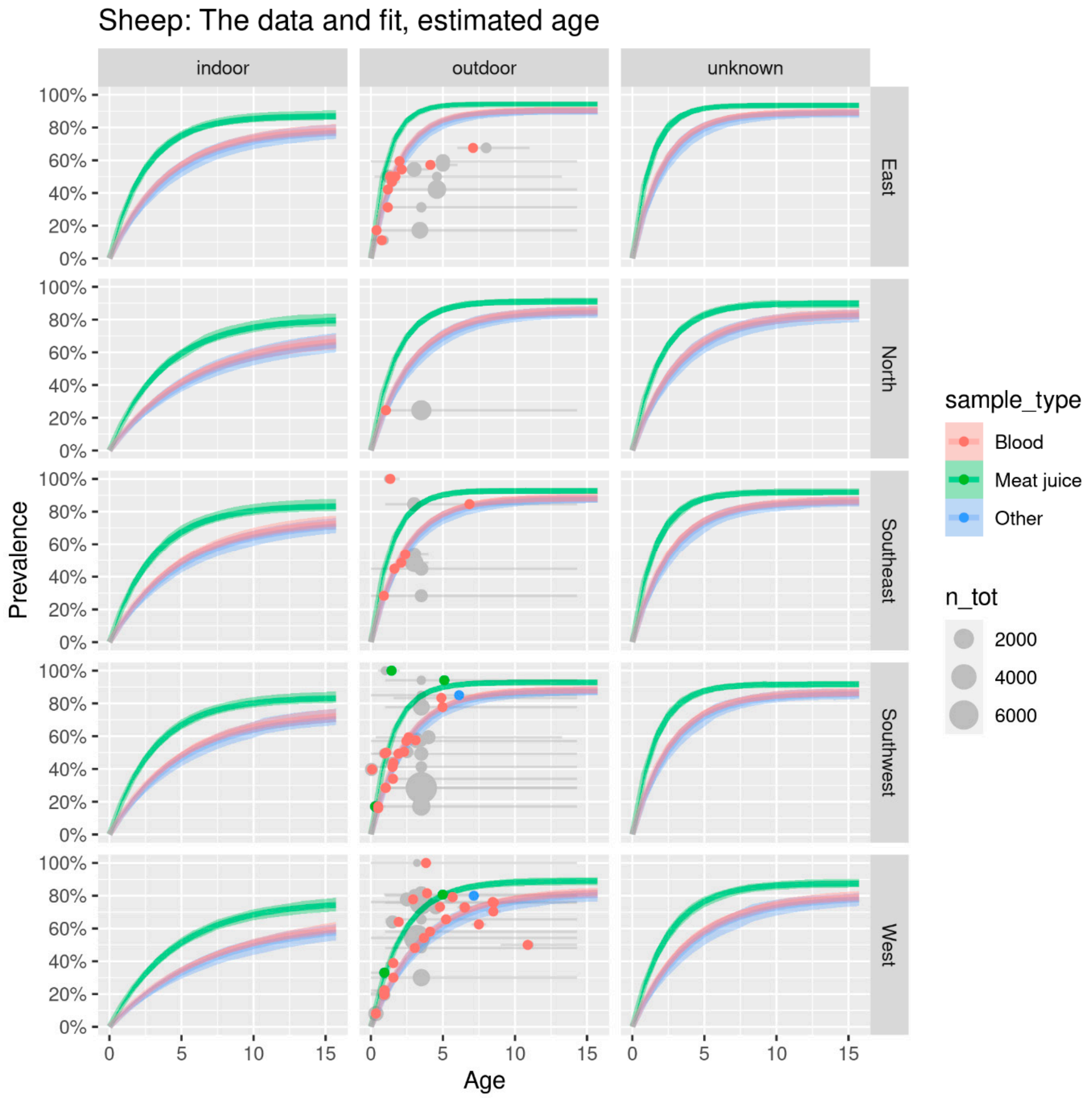

Figure S11: Modelled seroprevalence – sheep. SIS model fit for age-dependent seroprevalence of *T. gondii* in sheep by outdoor access in five regions of Europe. The lines indicate the fitted seroprevalence by age, based on detection in blood (red), muscle fluid (green) or other matrices (blue). The grey dots represent seroprevalence data points at the best estimate of age in the data for the studied populations. The dots are shifted horizontally along the grey line extending from the minimum to maximum possible age, to the best fitting age (red dots) (see Supplementary files – Fig. S15).

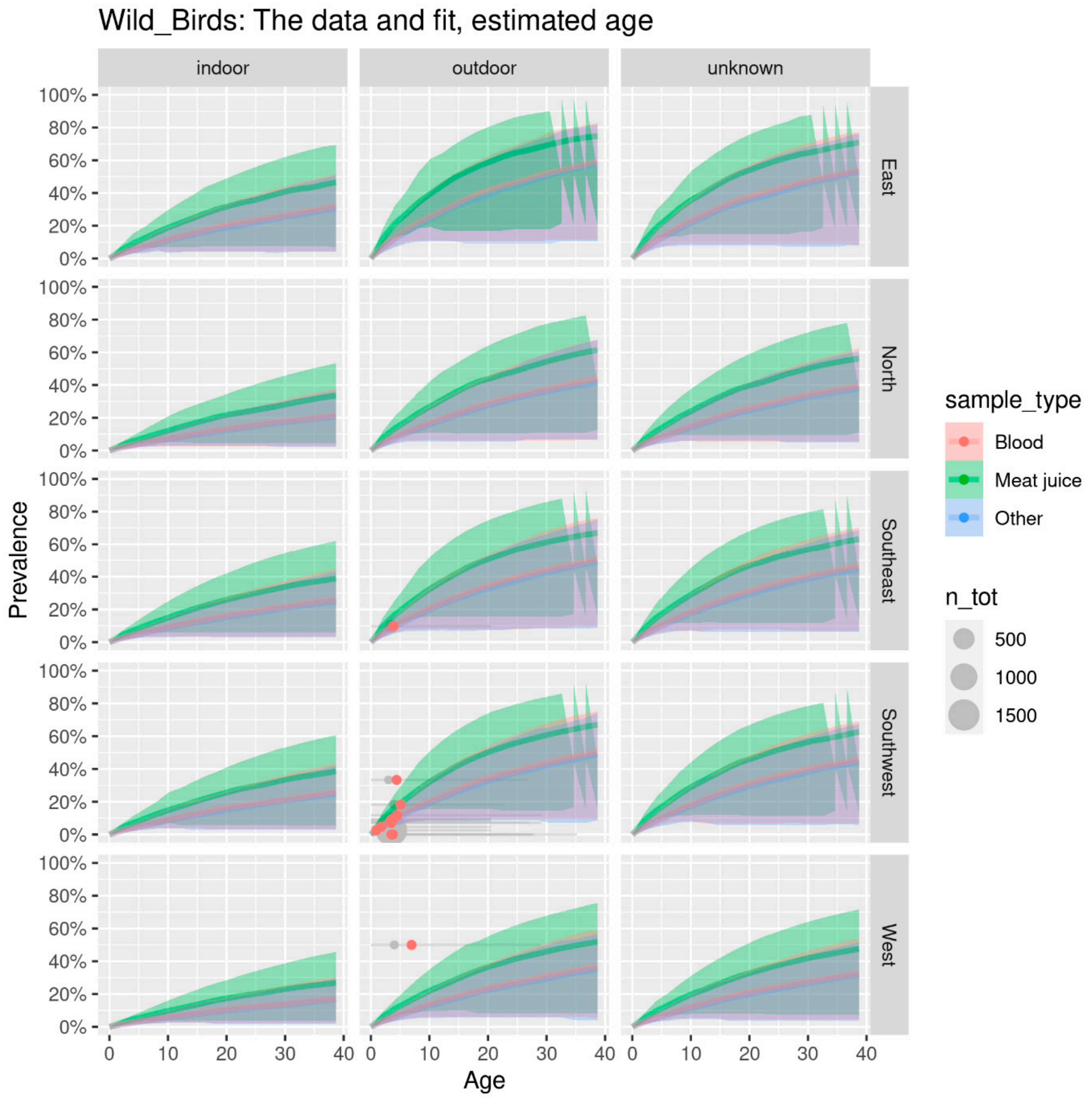

Figure S12: Modelled seroprevalence – wild birds. SIS model fit for age-dependent seroprevalence of *T. gondii* in wild birds by outdoor access in five regions of Europe. The lines indicate the fitted seroprevalence by age, based on detection in blood (red), muscle fluid (green) or other matrices (blue). The grey dots represent seroprevalence data points at the best estimate of age in the data for the studied populations. The dots are shifted horizontally along the grey line extending from the minimum to maximum possible age, to the best fitting age (red dots) (see Supplementary files – Fig. S15).

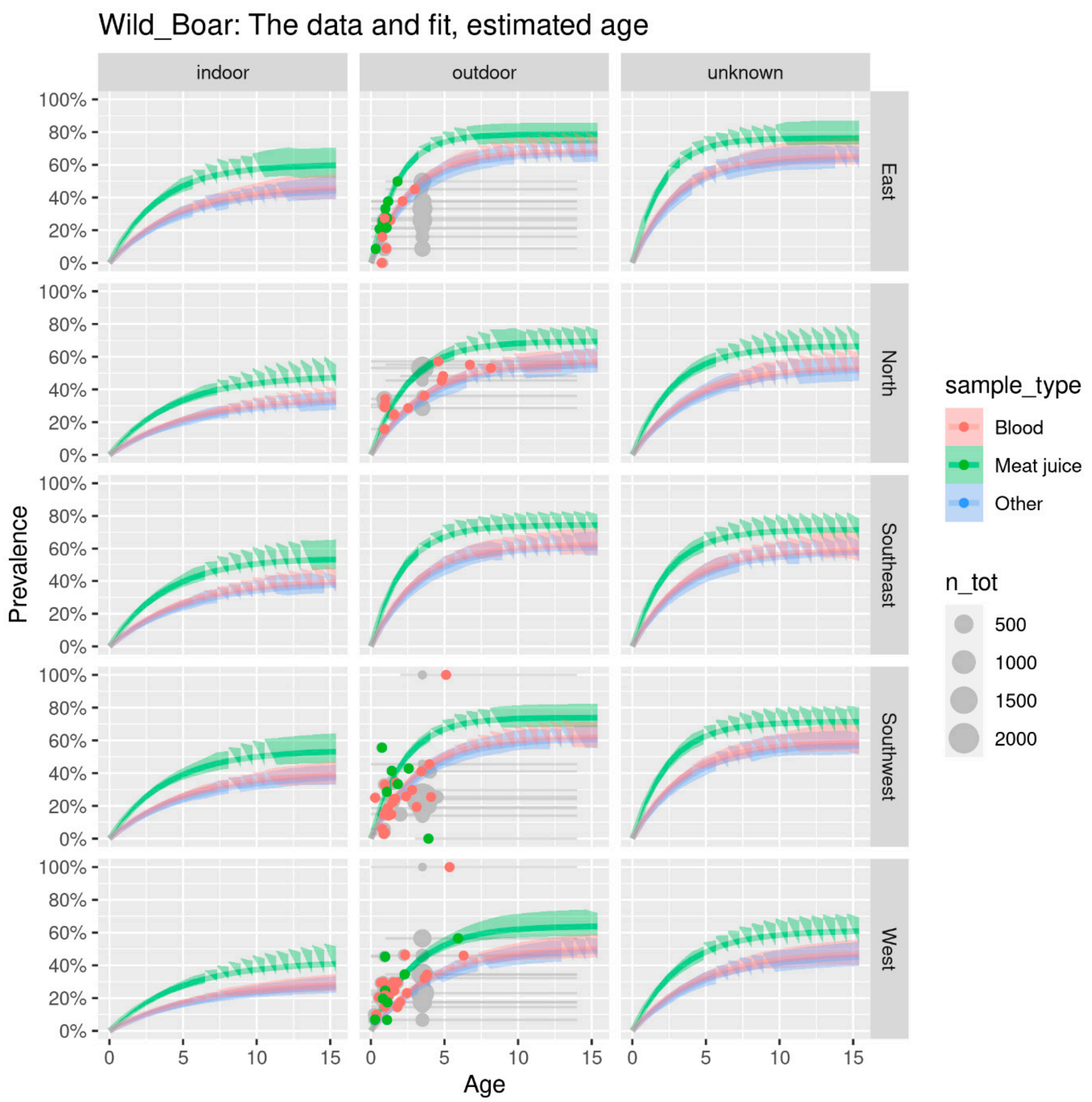

Figure S13: Modelled seroprevalence – wild boar. SIS model fit for age-dependent seroprevalence of *T. gondii* in wild boars by outdoor access in five regions of Europe. The lines indicate the fitted seroprevalence by age, based on detection in blood (red), muscle fluid (green) or other matrices (blue). The grey dots represent seroprevalence data points at the best estimate of age in the data for the studied populations. The dots are shifted horizontally along the grey line extending from the minimum to maximum possible age, to the best fitting age (red dots) (see Supplementary files – Fig. S15).

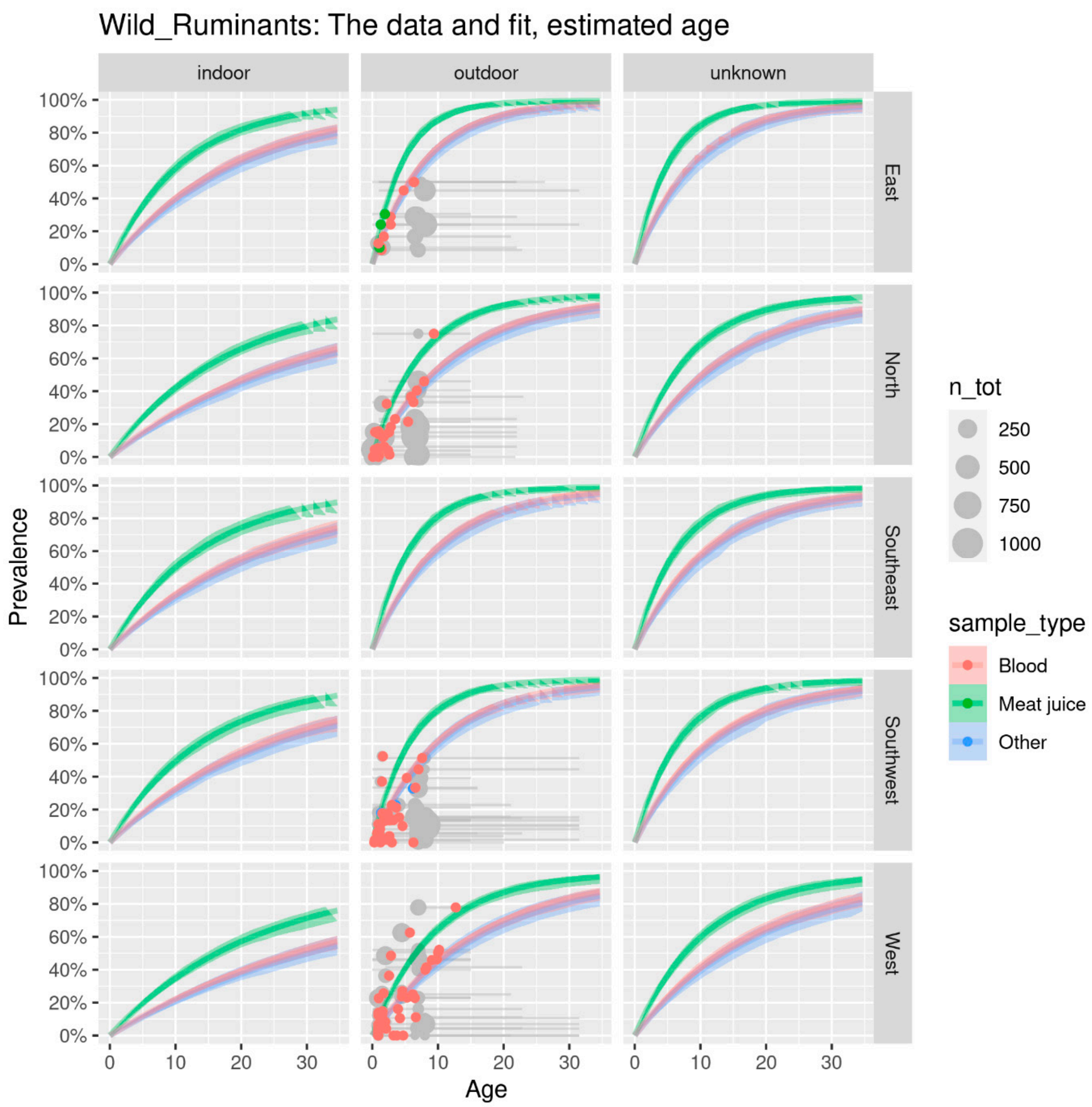

Figure S14: Modelled seroprevalence – wild ruminants. SIS model fit for age-dependent seroprevalence of *T. gondii* in wild ruminants by outdoor access in five regions of Europe. The lines indicate the fitted seroprevalence by age, based on detection in blood (red), muscle fluid (green) or other matrices (blue). The grey dots represent seroprevalence data points at the best estimate of age in the data for the studied populations. The dots are shifted horizontally along the grey line extending from the minimum to maximum possible age, to the best fitting age (red dots) (see Supplementary files – Fig. S15).

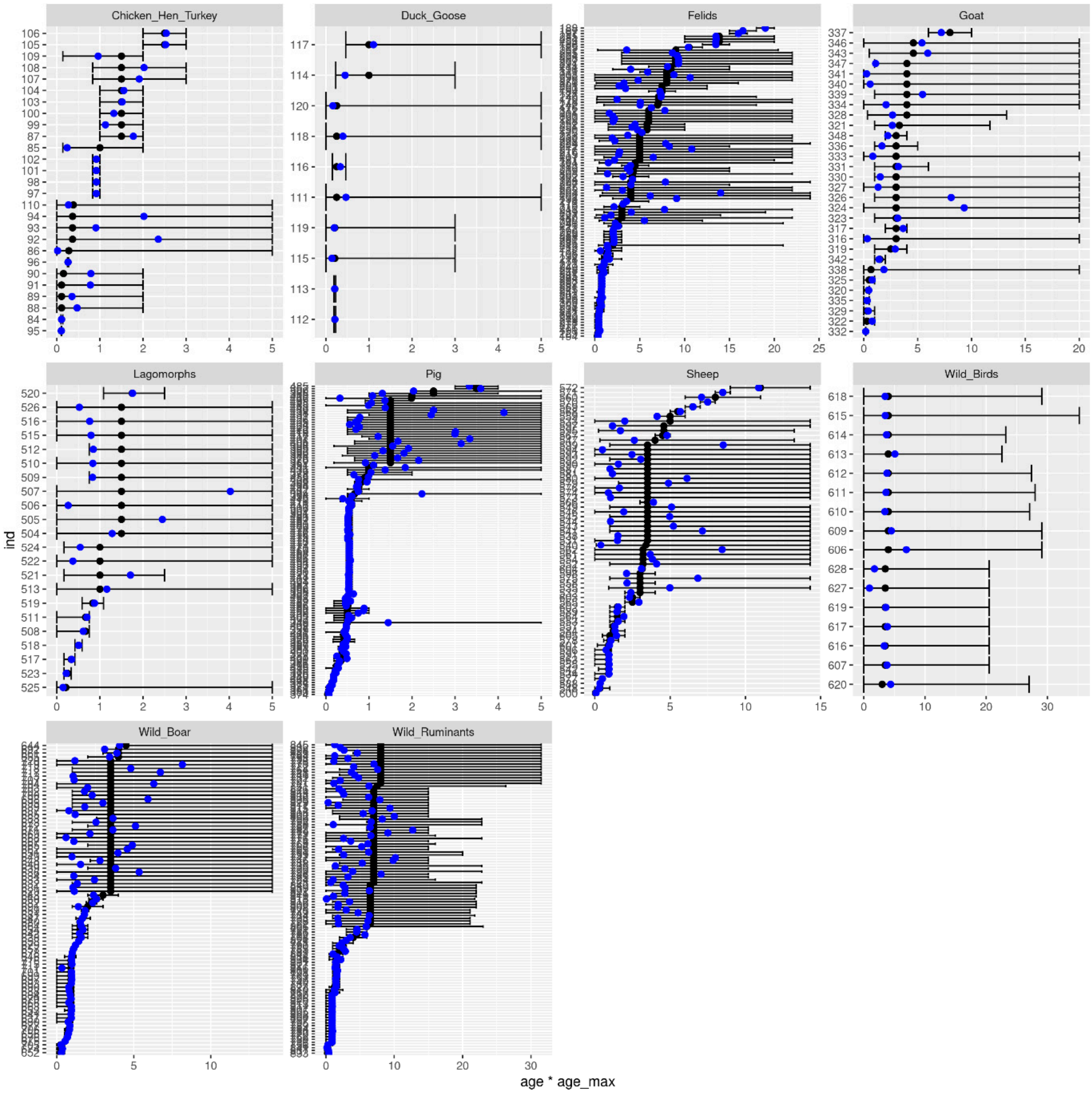

Figure S15: Age shift. The lines indicate the age range provided by publications and other sources as described as described in our study (see 2. Materials and methods). The black dots represent the most probable age at sampling input to the Bayesian hierarchical model. The black dots are shifted horizontally along the black line extending from the minimum to maximum possible age, to the best fitting age (blue dots) based on the SIS model.
