# Supplementary material for: Systematic Review and Modelling of Age-Dependent Prevalence of Toxoplasma gondii in Livestock, Wildlife and Felids in Europe
Source: Pathogens. 2023 Jan 6;12(1):97. doi: 10.3390/pathogens12010097 (PMC9865579; doi:10.3390/pathogens12010097)
Supplement: Supplementary file 1 [file pathogens-12-00097-s001.zip › Supplementary tables.pdf]

Supplementary tables to the article: "Systematic review and modelling of age-dependent prevalence of *Toxoplasma gondii* in livestock, wildlife and felids in Europe"

Content of the file: Table S1: Countries within European regions; Table S2: List of other matrices; Table S3: Number of included studies and articles per animal species; Table S4: Direct prevalence estimates.

Table S1: Countries within European regions. Categorization of European countries into five European regions – Wester, Northern, Eastern, Southeastern and Southwestern.

**Regions**	**Countries**
Western	Austria, France, Germany, Ireland, Lichtenstein, Luxembourg, the Netherlands, Switzerland, the United Kingdom
Northern	Denmark, Estonia, Finland, Iceland, Norway, Sweden
Eastern	Belarus, the Czech Republic, Hungary, Latvia, Lithuania, Poland, Romania, Slovakia, Ukraine
Southeastern	Albania, Bosnia and Herzegovina, Bulgaria, Croatia, Cyprus, Greece, Kosovo, Moldova, North Macedonia, Serbia, Slovenia
Southwestern	Andorra, Italy, Malta, Portugal, San Marino, Spain

Table S2: List of other matrices. List of "other" matrices, not belonging to "blood" or "meat juice", divided by the use of direct or indirect methods of detection. The names are provided as listed in included articles.

**Other matrices used with direct methods**	**Other matrices used with indirect methods**
Brain	Cardiac fluids
Diaphragm	Pleural fluids
Different tissues – heart, liver, mesenteric lymphnodes, mediastinal lymphnodes, diaphragm digest	
Heart	
Kidney	
Liver	
Milk	
Musculus masseter	
Organ	
Skeletal muscle	
Tissues	

Table S3: Number of included studies and articles per animal species. Publications are divided by the European region, testing method and animal species. The following format of "number of articles/number of animals" is used.

Animal species	Test method	East	Mix	North	Southeast	Southwest	West	Total
Buffalo	Direct	1/74	0/0	0/0	0/0	0/0	0/0	1/74
	Indirect	3/197	0/0	0/0	0/0	0/0	0/0	3/197
Cattle	Direct	2/163	1/151	0/0	0/0	0/0	9/1096	12/1410
	Indirect	19/12631	1/234	0/0	0/0	5/962	21/6118	46/19945
Felids	Direct	3/71	0/0	4/467	0/0	8/1428	15/104321	30/106287
	Indirect	13/1755	0/0	9/2426	0/0	45/3943	14/1987	81/10111
Goat	Direct	2/89	0/0	0/0	0/0	0/0	0/0	2/89
	Indirect	7/1011	0/0	1/2188	7/2107	8/3695	1/1664	24/10665
Equids	Direct	1/82	0/0	0/0	3/105	0/0	0/0	4/187
	Indirect	8/802	0/0	0/0	6/1230	16/1765	0/0	30/3797
Lagomorphs	Direct	0/0	0/0	0/0	1/52	1/144	1/57	3/253
	Indirect	5/2430	0/0	2/215	1/105	11/865	3/954	22/4569
Pig	Direct	7/1404	0/0	0/0	1/44	6/1122	5/385	19/2955
	Indirect	18/10633	0/0	6/3695	2/791	36/17658	23/16585	85/49362
Poultry	Direct	0/0	0/0	0/0	0/0	0/0	0/0	0/0
	Indirect	10/1307	0/0	0/0	0/0	4/653	17/6963	31/8923
Sheep	Direct	2/107	0/0	0/0	1/1	0/0	5/730	8/838
	Indirect	8/4975	0/0	1/1940	5/2849	18/15057	25/14206	57/39027
Wild birds	Direct	2/630	0/0	0/0	0/0	1/5	0/0	3/635
	Indirect	0/0	0/0	0/0	1/72	14/2562	1/4	16/2638
Wild boars	Direct	4/509	0/0	0/0	0/0	6/703	1/150	11/1362
	Indirect	15/3477	0/0	10/1899	0/0	26/4869	26/4719	77/14964
Wild ruminants	Direct	0/0	0/0	0/0	0/0	7/237	4/124	11/361
	Indirect	13/2014	0/0	25/6153	0/0	34/4569	37/2870	109/15606

Table S4: Direct prevalence estimates. Average weighted prevalence estimates for selected animal species, reported separately for different sample matrices used. Results of the meta-analysis for the results reported using direct methods with prevalence outcomes in % (with 95% CI in brackets) are sorted by region, animal species, sample type (matrix). Total number of animals for each animal category, region and sample type is represented in the column "n_tot".

Region	Species	Sample type	Prevalence	n_tot
East	Buffalo	Other	0.00% (0.00%,4.86%)	74
East	Cattle	Other	6.13% (2.98%,10.99%)	163
West	Cattle	Muscle	3.38% (2.17%,4.98%)	711
West	Cattle	Other	2.08% (0.90%,4.05%)	385
East	Equids	Other	0.00% (0.00%,4.40%)	82
Southeast	Equids	Other	1.90% (0.23%,6.71%)	105
East	Felids	Feces	1.41% (0.04%,7.60%)	71
North	Felids	Feces	0.17% (0.00%,0.93%)	598
Southwest	Felids	Feces	3.36% (2.49%,4.43%)	1429
Southwest	Felids	Other	6.67% (0.17%,31.95%)	15
West	Felids	Feces	0.15% (0.13%,0.18%)	104309
West	Felids	Other	33.33% (9.92%,65.11%)	12
East	Goat	Other	38.20% (28.10%,49.11%)	89
Southeast	Lagomorphs	Other	0.00% (0.00%,6.85%)	52
Southwest	Lagomorphs	Other	0.69% (0.02%,3.81%)	144
West	Lagomorphs	Other	68.42% (54.76%,80.09%)	57
East	Pig	Blood	25.53% (17.09%,35.57%)	94
East	Pig	Muscle	7.48% (6.11%,9.04%)	1310
Southeast	Pig	Muscle	0.00% (0.00%,8.04%)	44
Southwest	Pig	Muscle	19.61% (17.32%,22.05%)	1122
Southwest	Pig	Other	47.66% (37.92%,57.54%)	107
West	Pig	Muscle	5.97% (3.82%,8.83%)	385
East	Sheep	Blood	6.25% (2.06%,13.99%)	80
East	Sheep	Other	9.35% (4.57%,16.52%)	107
Southeast	Sheep	Other	33.33% (4.33%,77.72%)	6
West	Sheep	Muscle	8.19% (6.20%,10.58%)	647
West	Sheep	Other	3.61% (0.75%,10.20%)	83
East	Wild_Birds	Muscle	1.11% (0.45%,2.28%)	630
East	Wild_Birds	Other	2.38% (1.34%,3.90%)	630
Southwest	Wild_Birds	Other	0.00% (0.00%,52.18%)	5
East	Wild_Boar	Blood	4.42% (1.45%,10.02%)	113
East	Wild_Boar	Muscle	8.84% (6.23%,12.08%)	396
Southwest	Wild_Boar	Muscle	16.19% (9.72%,24.65%)	105
Southwest	Wild_Boar	Other	23.58% (20.23%,27.19%)	598
West	Wild_Boar	Muscle	0.67% (0.02%,3.66%)	150
Southwest	Wild_Ruminants	Muscle	1.92% (0.40%,5.52%)	156
Southwest	Wild_Ruminants	Other	22.90% (16.02%,31.05%)	131
West	Wild_Ruminants	Other	4.03% (1.32%,9.16%)	124
